# Bile Duct Injury During Laparoscopic Cholecystectomy: Has Anything Changed in 32 Years of Queensland Experience?

**DOI:** 10.7759/cureus.76216

**Published:** 2024-12-22

**Authors:** Bardia Bidarmaghz, Nestor Sabat, Peter Hodgkinson, Thomas O'Rourke, Nick Butler, Shinn Yeung, Kellee Slater

**Affiliations:** 1 Hepatopancreatobiliary Surgery, Princess Alexandra Hospital, Brisbane, AUS; 2 General Surgery, Mackay Base Hospital, Mackay, AUS

**Keywords:** bile duct injury, endoscopic retrograde cholangiopancreatography (ercp), intraoperative cholangiogram, laporoscopic cholecystectomy, roux-en-y hepaticojejunostomy, strasberg classification

## Abstract

Background

Bile duct injury (BDI) is a serious complication of laparoscopic cholecystectomy (LC). Large studies report an incidence of 0.08%-0.3%, but they also suggest that BDI in the LC era is more severe than in the era of open cholecystectomy. In light of our reported experience of managing BDI in 2002, this study aims to evaluate changes over the past two decades.

Methods

A single-center retrospective review for all patients referred to the hepatobiliary surgeons at the Princess Alexandra Hospital in Queensland, Australia for the management of BDI that occurred during LC from January 2001 to May 2022. This was compared to our historical data from 1990 to 2000 and statistically analyzed. Demographic characteristics, type of injury, intra-operative cholangiogram completion, attempted repair, the timing of referral to the tertiary center, and definite repair of BDI were analyzed.

Results

Sixty-five patients were referred to us with a similar severity of BDI to our previous study, but analysis showed an increase in intraoperative recognition of the injury to 74.4% (32 out of 43 patients). Additionally, the number of intra-operative cholangiograms performed increased dramatically to 66.2% (43 patients) which resulted in an increase in recognition of BDI. Conversion rate to open technique and attempted primary repair by operating surgeon decreased to 63% (27 patients) and 16% (11 patients), respectively, with referral time significantly shortened (P-value < 0.001).

Conclusion

The past two decades show an increased recognition of BDI, use of intra-operative cholangiogram, and decreased attempts to repair by the operating surgeon which can result in significant long-term complications. Instead, early recognition of BDI is critical for improved patient outcomes alongside expedited referral and appropriate surgical management by a hepatobiliary surgeon at a tertiary center.

## Introduction

Bile duct injury (BDI) is a serious complication of laparoscopic cholecystectomy (LC) and is associated with high morbidity and impaired quality of life [1,2]. The incidence of BDI after the introduction of LC was 1%-1.5%, and the high rate was attributed to the procedural learning curve [3]. Recent large series indicate that rates have been progressively decreasing and are now 0.08%-0.3% [4]. It would appear, however, that when BDI does occur, the injuries may be more severe than in the era of open cholecystectomy [5].

Injuries to the bile ducts range from bile leak from the cystic duct to complete transection of the major ducts accompanied by vascular injuries. The Strasberg classification with Bismuth modification is a generally accepted classification system for BDI. Several publications indicate that the best outcome following BDI will be achieved in a tertiary center with a multidisciplinary approach involving hepatopancreaticobiliary (HPB) surgeons, gastroenterologists, and interventional radiologists [6-8].

Our institution first reported its 10-year experience (1990-2000) in managing BDI in 2002 [9]. More than 20 years have passed since that report, and this study aims to evaluate any change over that time.

## Materials and methods

A single-center retrospective review was performed on all patients referred to Princess Alexandra Hospital (PAH) in Queensland, Australia, for the management of BDI during LC from January 2001 to May 2022. Approval for this study was given by Metro South Research Governance on September 11, 2023 (approval number: HREC/2022/QMS/88451).

Princess Alexandra Hospital is a major tertiary center located on the south side of Brisbane. It hosts a well-established HPB unit that accepts referrals from periphery hospitals across the state of Queensland. Patients in this cohort were treated by one of six HPB surgeons. Data for this review were collected from hospital electronic medical records and the operation theatre database. The analysis focused on patient demographics (age, gender), recognition of the injury by the primary surgeon during the LC, use of intraoperative cholangiogram (IOC) and its role in diagnosing the injury, the time between referral and definite repair at PAH, and postoperative complications. Additionally, the decision-making process of the primary surgeon was evaluated, including whether the operation was converted to open surgery and whether an attempt was made to repair the injury. The type of injury was recorded according to the Strasberg-Bismuth Classification [10]. All definitive surgeries were performed by an HPB surgeon.

The outcomes of patients treated between 2001 and 2022 were compared to a previously published cohort from this hospital, reported by Slater et al., covering the years 100- to 2000 [9]. Statistical analysis, including Fisher’s exact test, was performed to identify changes in the management of BDI over the past 32 years.

Patients with type A injuries (cystic duct leakage) managed conservatively with endoscopic retrograde cholangiopancreatography (ERCP) and stenting, as well as those with concurrent non-bilioarterial injuries, were excluded from the study.

## Results

Demographics

Between 2001 and 2022, 65 patients were referred to the PAH for the management of iatrogenic BDI occurring during LC. This compares favorably to 58 patients referred over the preceding ten-year period (1990-2000). In the current cohort, there were 42 females and 23 males with a mean age of 61.6 (age range 22-80). 

**Figure 1:**
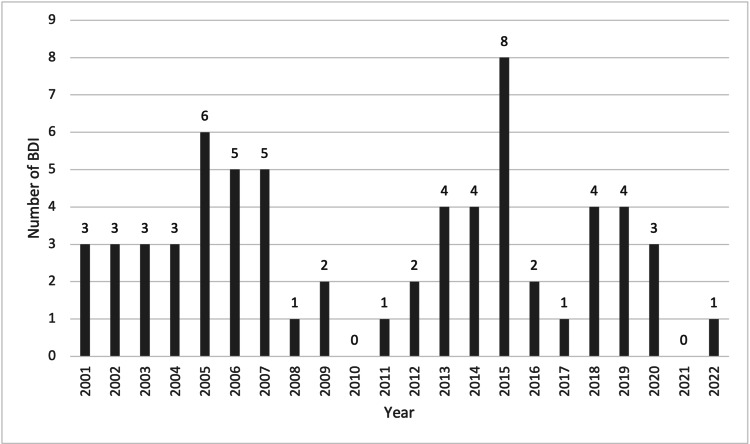
Number of BDI repaired per year 2001–2022. There was zero referral to our center in 2021 due to decreased number of elective laparoscopic cholecystectomy during the COVID-19 pandemic.

Three out of 65 patients were referred from centers outside of Queensland, and two patients sustained their injury at the PA hospital during an LC performed by a surgical subspecialty other than the HPB team. The years in which the BDI repair was performed are outlined in Figure 1.

Injury recognized intra-operatively

The BDI was recognized intraoperatively in 43 patients (66%). Sixty-three percent (27/43) were converted to an open operation, and this was far fewer than in the earlier series, where 92% (24/26) of patients were opened when a BDI was identified.

From 2001 to 2022, an attempt at primary repair by the referring surgeon was performed in only 11 (16%) patients, compared to 80% in the earlier series. In the other 32 patients where the injury was recognized, instead of attempting a primary repair, intraoperative advice was sought from the HPB unit by the referring surgeon. In nine patients, an HPB surgeon attended the referring hospital while the patient was still under anesthesia. The remaining patients had an abdominal drain inserted close to the injury and were transferred to our facility for definitive management.

Injury not recognized intra-operatively

In addition, in the recent series, there were 22 (33%) patients whose BDI was unrecognized intraoperatively, a decrease from the 32 (55%) patients reported in the previous study. Eleven (50%) of these patients presented with symptoms of biliary peritonitis between three and 60 days postoperatively and were referred to our center. The remainder 11 (50%) of patients presented with painless obstructive jaundice. 

For the patients with delayed recognition of BDI, laparoscopic washout, and drain insertion occurred in 15 (68%) patients prior to transfer to our center. While the remaining seven (32%) patients were transferred after leaking bile into their intraoperatively placed drains.

Intra-operative cholangiogram

From 2001 to 2022, 66.2% (43/65) of patients had IOC performed at the time of LC compared with only 36.2% (21/58) in the earlier series, which was statistically significant. Of those patients who had an IOC performed, 74.4% (32/43) in the current series had their BDI identified intraoperatively compared to only 33% (7/21) in the earlier series and this was also statistically significant. Additionally, when no IOC was performed during LC, only 17% of BDI injuries were identified in the recent series, down from 33% in the earlier study (p=0.41) (Table 1).

**Table 1:** BDI data for the decade of 1990–2000 and the 22 years between 2001 and 2022. *Interpretation of IOC by the primary surgeon **Primary suture repair, T-tube insertion, or duct-to-duct repair Two-tailed P-values were calculated using the Fisher Exact Test

	1990–2000	2001–2022	P-value
Number of injuries	58	65	-
Number of females	46 (79.3%)	42 (64.6%)	0.0714
Mean age (SD)	50.1 (20)	61.1 (18.3)	0.0012
Injury recognized intra-operatively	26 (44.8%)	43 (66.2%)	0.0174
Cholangiogram performed	21 (36.2%)	43 (66.2%)	<0.001
Cholangiogram recognized injury*	7 (33.3%)	32 (74.4%)	<0.001
Cholangiogram not performed	37 (63.8%)	22 (33.8%)	<0.001
Cholangiogram not performed and BDI not recognized	19 (51%)	11 (50%)	0.041
Primary surgeon converted to open	24 (92%)	27 (63%)	0.984
Primary surgeon attempted repair**	21 (80%)	11 (16%)	0.0151
Referral < 14 days to HPB unit	22 (37.9%)	56 (86.2%)	<0.001

Type of injury

The injuries sustained at LC are depicted in Table 2. The sustained injuries, including the more severe class E injuries, were not statistically different between the two groups. Comparing the frequency and type of injury sustained during LC in this study compared to the study completed in 2002 showed no statistically significant difference between the type of injury sustained between these two time periods during LC. Thirteen (20%) patients in the current series had a concomitant hepatic artery injury compared to 14 (24%) in the earlier series.

**Table 2:** Summary of all the BDI during 1990–2000 and 2001–2022, classified according to the Strasberg classification of bile duct injury. Two-tailed P-values were calculated using the Fisher Exact Test

Strasberg injury classification	1990-2000 (n=58)	2001-2022 (n=65)	P-value
B	3 (5%)	0	0.063
C	4 (6.8%)	10 (6.5%)	0.139
D	6 (10%)	15 (23%)	0.061
E1	11 (18.9%)	3 (4.6%)	0.012
E2	17(29%)	18 (27.6%)	0.841
E3	5 (8.6%)	13 (20%)	0.075
E4	8 (13.7%)	3 (4.6%)	0.075
E5	4 (6.9%)	3 (4.6%)	0.582

Surgical management of BDI

HPB surgeons at the PAH in the current series repaired the BDI using a Roux-en-Y hepatico/choledochojejunostomy appropriate for the type of injury identified by preoperative imaging, direct cholangiography, and dissection during definitive surgery. 

Six patients required a partial hepatectomy (five right, one left, one segmental resection) for haemobilia not responsive to angiographic treatment, for hepatic duct stricturing not amenable to reconstruction, to improve access for biliary reconstruction. The number of bile ducts reconstructed and whether concomitant hepatectomy was performed are outlined in Table 3, comparing the two time periods. 

**Table 3:** Summary of the types of BDI repairs undertaken for the periods of 1990 - 2000 and 2001 – 2022. Tow-tailed P values were calculated using Fisher Exact Test

Repair type	1990 – 2000	2001 – 2022	P value
Choledochojejunostomy one anastomosis	38 (65.5%)	51 (78.5%)	0.11
Hepaticojejunostomy > one duct	10 (17.2%)	7 (10.8%)	0.31
Hepaticojejunostomy + hepatectomy	5 (8.6%)	6 (9.2%)	0.91

Complications

In the current series, following Roux-en-Y reconstruction, only 16 (24.6%) patients were diagnosed with postoperative complications; however, all were expeditiously managed by the HPB team without long-term complications. There were no deaths attributable to BDI in the time period.

Ten patients (15%) presented with ascending cholangitis between one and three years postoperatively and developed an anastomotic stricture. Two of these patients underwent therapeutic dilatation of the stricture using a percutaneous transhepatic route. Three patients required operative revision of the Roux-en-Y choledochojejunostomy for strictures that were not dilatable. The remaining patients were treated with antibiotics and imaging did not reveal any significant biliary stricture.

One patient underwent hepaticojejunostomy for a right posterior segmental duct occluded by an LC clip three years later, which was not identified at the first repair. One patient required delayed hemi-hepatectomy following Roux-en-Y repair for type E BDI, due to severe, unreconstructed stricturing of the right hepatic duct.

Two patients developed intrabdominal abscesses in the postoperative period with one drained under imaging guidance while the other required a re-look laparotomy and drainage of a liver abscess. Finally, four patients had a minor bile leak managed with an intraoperatively placed drain with full patient recovery.

In the first series, 92% of the patients recovered without complications at the time of the publication. However, owing to the loss of follow-up of these patients, it is likely that postoperative complications related to long-term biliary strictures may be underdiagnosed.

## Discussion

Approximately 20,000 LCs are performed each year in the state of Queensland across public and private healthcare systems [11,12]. This three-decade-long study encompasses and reflects on the rise and fall of the LC “learning curve” in Australia. Multiple large international population-based studies have concluded that the incidence of BDI has become static, occurring at rates of 0.32% to 0.8% [13-17]. While our data may infer a slight decrease in the number of referrals between the two time periods, this cannot be assumed, as alternate referral pathways have evolved in our state due to the gradual increase in the number of hospitals employing specialist HPB surgeons.

When BDI occurs during an LC, early recognition followed by expedited and appropriate surgical management is critical. Multiple studies have shown better outcomes, including long-term morbidity, mortality, and improved quality of life, in patients who underwent immediate repair of their BDI [13,18-20].

Over the two time periods, the actions of the referring surgeon when recognizing an injury during LC have significantly changed. The first change is that they are converting to laparotomy far less frequently. It is likely that surgeons have become more unfamiliar with the operation of open cholecystectomy and that this may make an already difficult operation even more complicated. 

The second change is that surgeons recognize the role of specialist center involvement in achieving the best outcomes for these patients. A referring surgeons’ judgment may likely be clouded due to the anxiety created by injuring a bile duct. It is always a good practice to consult a colleague when BDI has been recognized. Halle-Smith et al. showed a four-time risk of postoperative complications if the BDI was repaired by a non-HPB surgeon [21].

In the first series, 80% of the surgeons attempted to repair the injury. Now, the majority of surgeons are calling for advice during the case and referring the patient for definitive repair. Management decisions following BDI are significantly reliant on the surgeon’s experience, which has important short- and long-term implications for patient outcomes [22].

Immediate management of BDI may require a variety of techniques, including endoscopic or percutaneous stenting and operative repair. Whilst many centres are embracing multiple endoscopic stenting procedures, mainly for Type D injuries, operative management has remained the definitive treatment at this center. Wang et al showed that immediate surgery performed by an HPB surgeon has a significantly lower failure rate (18.9%) compared to the overall immediate repair rate (60%) that included all general surgeons [23]. Hepaticojejunostomy using the Hepp-Couinaud technique is the gold standard repair technique for most cases of BDI [17,24]. It is not, however, an operation that surgeons working outside a specialist unit frequently encounter. In particular, biliary-enteric anastomosis of the non-dilated bile ducts is a complex procedure. The performance of this operation by the referring surgeon, who may be stressed as a result of causing the injury, poses a significant risk of biliary leak or long-term anastomotic strictures [25]. However, in the hands of HPB surgeons, this is a routine procedure, often encountered in the daily management of adult and pediatric cases for a multitude of benign and malignant conditions, including liver transplantation [17,26].

The state of Queensland is geographically expansive, with many regional centers lacking onsite HPB surgeons experienced in the management of BDI. Our tertiary facility operates a 24-hour-a-day service to offer advice on bridging strategies to manage patients where BDI has been recognized intra-operatively. If the patient is under anesthesia within a reasonable driving distance of our facility, one of the HPB surgeons will attend the referring hospital and repair the patient’s BDI at the time of injury. For patients where local travel by the HPB team is not possible, management usually involves laparoscopic drain placement without conversion to open operation to allow for expedient patient transfer to our facility by fixed-wing aircraft. The wide advertisement of this service has significantly increased the early referral of patients in Queensland, and according to our results, has resulted in expedited definitive management. 

The optimal timing of repair following delayed presentation of BDI is less clear in the literature, with patients undergoing repair when a bile leak and biliary peritonitis have occurred experiencing the least favorable outcomes [27]. Presentation of patients more than two weeks following their BDI significantly dropped between the two series we present here. When the presentation is delayed, management becomes even more complex, and the literature strongly suggests that BDI repair in these patients should be attempted only by specialist surgeons with adequate training in bilioenteric reconstruction [22]. Safe transfer requires stabilization of the patient often with endoscopic and/or operative washout and drainage. A large meta-analysis suggested that it is best to avoid repair in patients with delayed presentations until approximately six weeks after the initial injury [28].

Intraoperative cholangiography is a valuable tool routinely used during LC to identify common bile duct stones, define surgical anatomy, and recognize BDI [19]. The number of patients undergoing routine IOC increased in our latter series and this was statistically significant, as was the number of bile duct injuries recognized by the IOC. However, there is room for improvement in the interpretation of the IOC. 21% of patients who had completed IOCs still did not have their BDI recognized. It is possible of course, that the injury occurred following the completion of the IOC. Surgeons’ interpretation of an IOC may be hampered for many reasons: inadequate filling of the upper ducts or failure to recognize “missing” ducts that are unopacified due to occlusion or injury are common failures. Ongoing training in the interpretation of IOC is very important.

Complications in this study were those expected following a Roux-en-y biliary reconstruction and the rates are consistent with the literature. Anastomotic leaks in the immediate postoperative period have been reported at a rate of 3.7%. Our reported biliary stricture rates and management were also consistent with those of other studies [29].

More than 20% of patients in both series sustained hepatic artery injury in conjunction with the BDI. Ensuring biliary ischemia is likely to contribute to the higher rate of biliary stricture in these patients. We recognize the significant limitations of comparing long-term complications in this series. The vast majority of patients in the first series are now lost to follow-up; many are deceased and the lack of a digital record for that time period, makes tracing their clinical course impossible. 

## Conclusions

BDI during LC is a serious complication that needs careful management and planning. We report a comparison of this HPB center’s early and more recent experience in referral patterns and management of BDI. Between the two periods, we noted a significant increase in the recognition of the BDI during the LC and an increase in the immediate referral of patients for tertiary care. There was a reduction in conversion to an open procedure and a decrease in the number of surgeons attempting to repair the injury themselves. The severity of the injuries has not changed over time; however, there has been a significant increase in the number of IOCs being performed during the LC.
